# Supplementary material for: PLOD2 Is a Prognostic Marker in Glioblastoma That Modulates the Immune Microenvironment and Tumor Progression
Source: Int J Mol Sci. 2022 May 27;23(11):6037. doi: 10.3390/ijms23116037 (PMC9181500; doi:10.3390/ijms23116037)
Supplement: Supplementary file 1 [file ijms-23-06037-s001.zip › ijms-1704782-supplementary.pdf]

Supplementary Figure S1

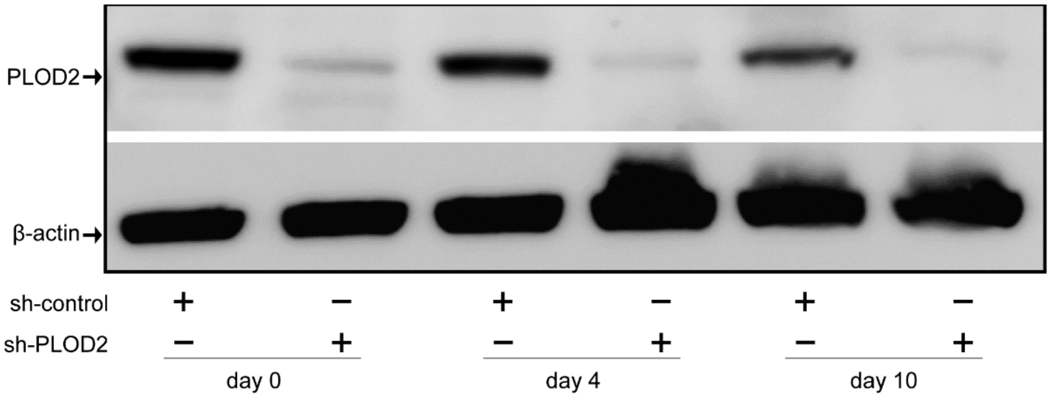

Supplementary Figure S1. Stability of PLOD2 knock-down during functional assays. Transfected H4 cells were cultured in medium without puromycin. The cells were lysed at day 0, day 4 and day 10 in culture and PLOD2 expression was analysed by western blot. Beta-actin was used as loading control.

Supplementary Figure S2

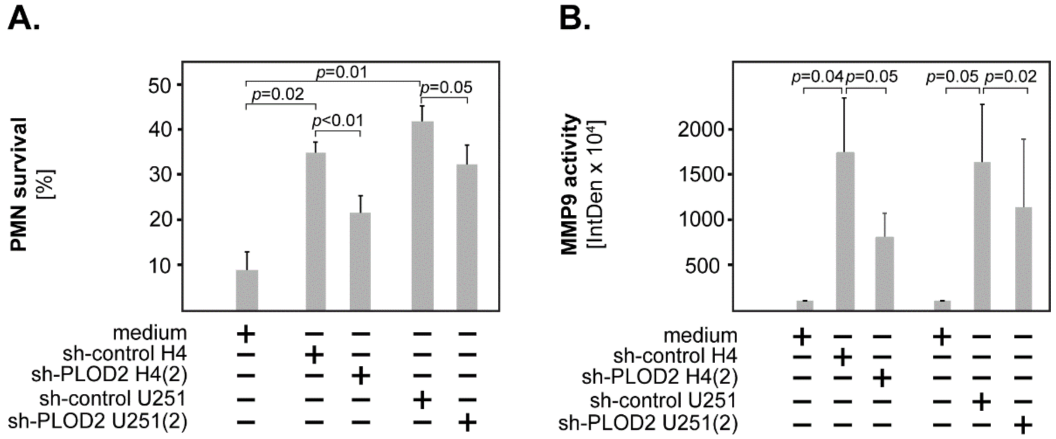

Supplementary Figure S2. PLOD2 and neutrophils in GBM – exclusion of clonal effects. Neutrophils were incubated with supernatants (SN) derived from sh-control or a second clone (2) of sh-PLOD2 GBM cells. Culture medium was used as control. The survival of neutrophils was assessed at 24 h and the release of MMP9 at 1 h post-stimulation. **(A)** Sh-control SN prolonged the survival of neutrophils in both cell lines. This effect was significantly lower upon stimulation with PLOD2 knock-down SN **(B)** Neutrophils stimulated with sh-PLOD2 SN released significantly lower levels of MMP9 compared with their sh-control stimulated counterparts. For quantification, the integrated density of the bands was determined using the ImageJ software. The data are presented as percentage to medium only. Shown are the means + S.D. of three independent experiments. Statistical analysis was performed with the paired *t*-test.

Supplementary Figure S3

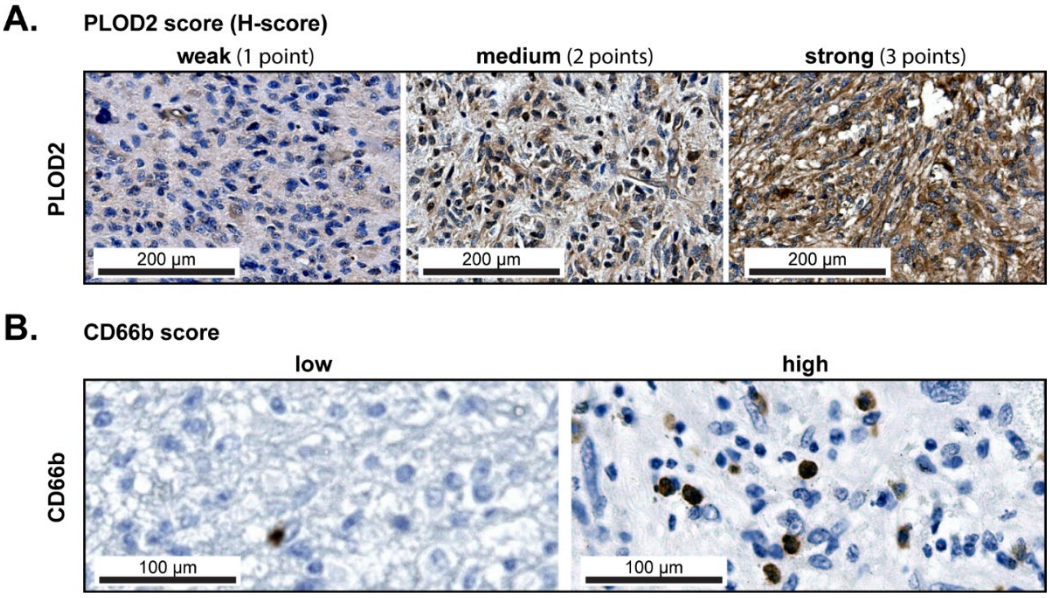

Supplementary Figure S3. PLOD2 and CD66b: expression and scoring in GBM tissues. (A) Representative micrographs showing "weak" (1 point), "medium" (2 points) and "strong" (3 points) expression of PLOD2 in GBM tissues. The H-score was subsequently calculated according to the formula: (1 x X) + (2 x Y) + (3 x Z), where X + Y + Z = 100% of the total tumor area. **(B)** Representative micrographs showing low and high CD66b expression in GBM tissues.

Supplementary Figure S4

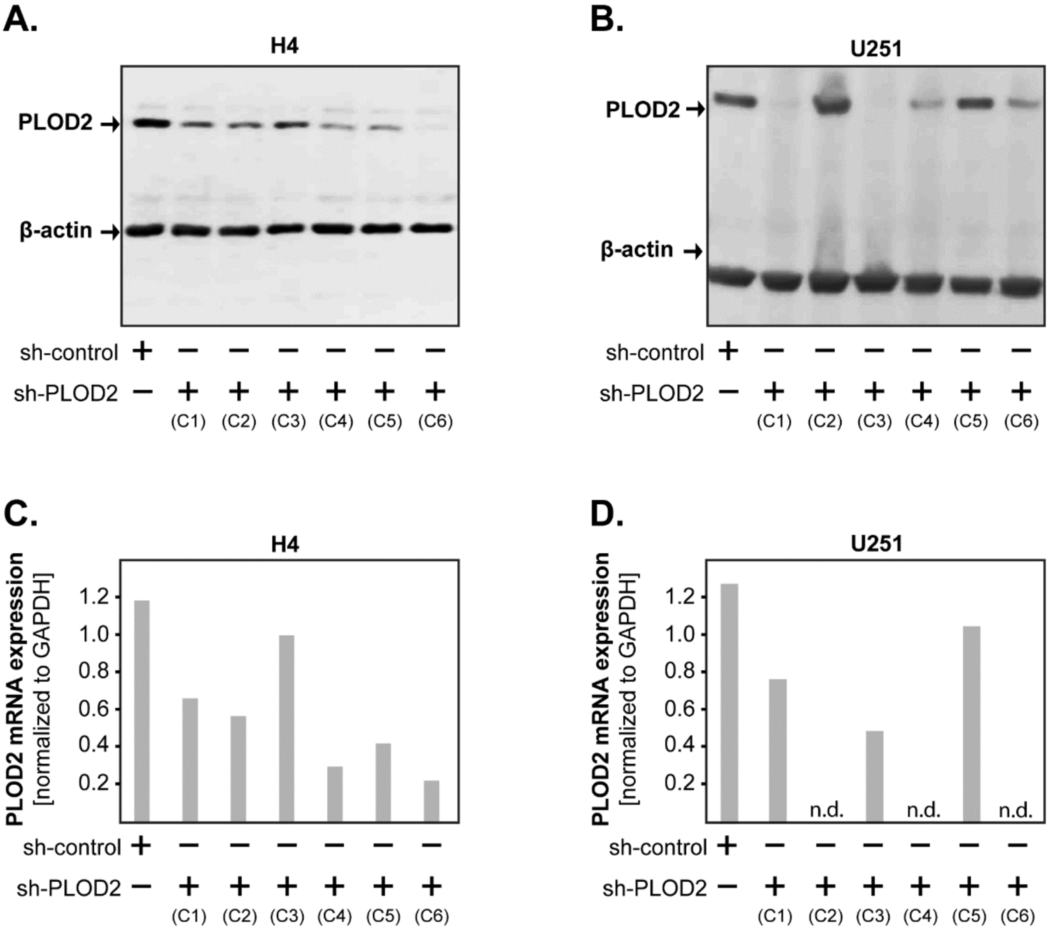

Supplementary Figure S4. Stable knock-down of PLOD2 in H4 and U251 GBM cell lines. H4 and U251 cells were transfected with PLOD2 shRNA (sh-PLOD2) or a control shRNA (sh-control). After antibiotic selection and limiting dilution, we tested 6 clones (C1-C6) for PLOD2 expression. Representative western blot showing PLOD2 expression in sh-control versus sh-PLOD2 C1-C6 in **(A)** H4 cells and **(B)** U251 cells. Beta-actin was used as loading control. PLOD2 mRNA levels in knock-down versus control **(C)** H4 cells and **(D)** U251 cells. GAPDH was used as a housekeeping gene to normalize the expression of PLOD2 in these cells. Clone C6 (H4 cells) and clone C3 (U251 cells) were used in all experiments. Clone C4 (H4 cells) and clone C1 (U251 cells) were used in selected experiments to exclude clonal effects.

Supplementary Table S1

	Hannover cohort				Magdeburg cohort			
	all patients		IDH WT		all patients		IDH WT	
	No.	%	No.	%	No.	%	No.	%
**All patients**	204	100	181	100	203	100	164	100
**Sex**								
female	87	42.6	76	42.0	95	46.8	78	47.6
male	117	57.4	105	58.0	108	53.2	86	52.4
**KPS**								
0	0	0	0	0	3	1.5	3	1.8
10	2	1.0	2	1.1	0	0	0	0
20	1	0.5	1	0.6	2	1.0	2	1.2
30	3	1.5	2	1.1	9	4.4	9	5.5
40	10	4.9	9	5.0	10	4.9	8	4.9
50	26	12.7	23	12.7	32	15.8	29	17.7
60	43	21.1	39	21.5	38	18.7	27	16.5
70	40	19.6	37	20.4	70	34.5	54	32.9
80	41	20.1	36	19.9	19	9.4	16	9.8
90	29	14.2	23	12.7	19	9.4	15	9.1
100	1	0.5	1	0.6	0	0	0	0
n.d.	8	3.9	8	4.4	1	0.5	1	0.6
**Therapy**								
surgery	22	10.8	22	12.2	36	17.7	31	18.9
surgery + RTX	26	12.7	25	13.8	21	10.3	18	11.0
surgery + CTX	5	2.5	4	2.2	1	0.5	1	0.6
surgery + RCTX	140	68.6	121	66.9	140	69.0	109	66.5
n.d.	11	5.4	9	5.0	5	2.5	5	3.0
**Surgical resection**								
total	81	39.7	68	37.6	77	37.9	59	36.0
subtotal/biopsy	110	53.9	101	55.8	115	56.7	96	58.5
n.d.	13	6.4	12	6.6	11	5.4	9	5.5
**MGMT status**								
methylated	90	44.1	79	43.6	118	58.1	100	61.0
unmethylated	99	48.5	87	48.1	78	38.4	64	39.0
n.d.	15	7.4	15	8.3	7	3.4	0	0
**IDH mutation**								
wild-type	181	88.7	181	100	164	80.8	164	100
mutated	9	4.4	0	0	10	4.9	0	0
n.d.	14	6.9	0	0	29	14.3	0	0

Supplementary Table S1. Clinical characteristics of GBM patients. WT: wild-type; KPS: Karfnofsky Performance Scale; RTX: radiotherapy; CTX: chemotherapy; RCTX: radio-chemotherapy; n.d.: not determinable.

Supplementary Table S2

	H4 cells		U251-MG cells	
	**Properties**	**Source**	**Properties**	**Source**
**Age**	37	Expasy; ATCC	75	Expasy; DOI:10.1002/cam4.219
**Sex**	male		male	
**Ethnicity**	caucasian		caucasian	
**Morphology**	epithelial		epithelial	
**Growth**	adherent		adherent	
**Karyotype**	hypertriploid 73, XXYY in 26% of cells		DNA index 1.75; 66, XXY	
**PTEN**	mutation (missense)	DOI: 10.1023/a:1010680920087; DepMap portal	mutation (frameshift)	DOI: 10.1111/j.1750-3639.1999.tb00536; DepMap portal
**TP53**	LOH mutation	DOI: 10.1023/a:1010680920087	mutation	DOI: 10.1111/j.1750-3639.1999.tb00536
**CDK2NA/ p16**	no mutation	DOI: 10.1186/1756-9966-30-76	deletion	DOI: 10.1111/j.1750-3639.1999.tb00536
**EGFR**	low expression	DOI:10.1007/s11060-018-2903-8	moderate expression	DOI: 10.1007/s11060-018-2903-8

Supplementary Table S2. Characteristics of the GBM cell lines used in this study.
